# Reply: The true and false aortic annulus?

**DOI:** 10.1016/j.xjtc.2023.12.002

**Published:** 2023-12-13

**Authors:** Bo Yang

**Affiliations:** Department of Cardiac Surgery, University of Michigan, Ann Arbor, Mich

Reply to the Editor:

**Figure:**
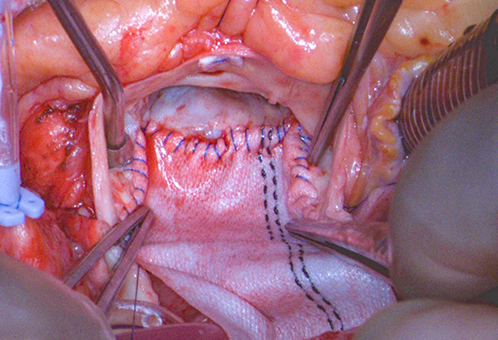
The Y-AAE enlarges the surgical aortic annulus/root by enlarging the aortomitral curtain.

There has been criticism since the Y-incision aortic annular enlargement (Y-AAE) was published in 2021 that the Y-AAE does not enlarge the basal ring—the true aortic annulus—as in the traditional aortic annular enlargement techniques, including Manougian and Konno procedures. Lick^1^ raised the same concern in their letter in this issue of *JTCVS Techniques*. The debate boils down to 2 questions: Do we need to enlarge the basal ring for AAE during aortic valve replacement (AVR) for patients with aortic valve pathology only, such as aortic stenosis or insufficiency? and, If the Y-AAE does not enlarge the basal ring (the true aortic annulus as stated by Lick^1^), what aortic annulus does this technique enlarge, a false aortic annulus?

We responded to a similar question in our response in *JTCVS Techniques* previously.^2^ Surgeons do not need to enlarge the basal ring or left ventricular outflow tract (LVOT) for AVR in adult patients with only aortic valve pathology. We strongly recommend surgeons who have similar questions read that article.^2^ In patients with aortic valve pathology only, the size of basal ring and LVOT are normal as seen in preoperative and postoperative echocardiogram in our recent study^3^ but the aortic valve is stenotic or incompetent. For AVR, surgeons do not suture the prosthetic valve at the basal ring—an imaginary ring.^4^ The traditional techniques, such as Manougian and Konno procedures, incise deep into the LVOT to further enlarge the surgical aortic annulus/root above basal ring to accommodate a larger valve but not for the purpose of enlarging the basal ring and LVOT per se since they are normal in those patients. In patients with aortic stenosis and subannular web or hypertrophic obstructive cardiomyopathy, resection of the subannular web or myectomy plus Y-AAE AVR is recommended. In congenital subaortic tunnel or hypoplastic LVOT, the Konno procedure is recommended. In our recent study,^3^ we found with Y-AAE alone in 119 consecutive patients (67% women, mean body mass index of 30, median annular size of 21 mm, median prosthetic valve size of 29), the LVOT is enlarged significantly by 10% and the mean gradient across the LVOT is 2 mm Hg before and after AVR. The valve area continuously increased to 2.3 cm^2^ and mean gradient across the prosthetic valve remains 7 mm Hg 2 years after the operation. Because Y-AAE does not disturb the mitral valve, the mitral function remained normal or improved after the operation.^3^

What does Y-AAE enlarge? Is there a false aortic annulus? The Y-AAE enlarges the surgical aortic annulus (or the anatomic aortic annulus) and aortic root by enlarging the aortomitral curtain (Figure 1). The surgical aortic annulus is the crown-shape aortic annulus where the aortic cusps attach to the aorta, which only surgeons can see to suture the prosthetic valve to. The basal ring does not attach to the new prosthesis in all areas, as Lick^1^ stated in their letter, but only at 3 points (3 nadirs of the aortic annulus). With Y-AAE enlarging the surgical aortic annulus, we have been able to consistently upsize 3 to 4 valve sizes routinely in 126 consecutive cases to date.^3^^,^^4^ Compared with patients treated with a Nicks or Manougian procedure, we find the hemodynamics in patients treated with Y-AAE are significantly better, including mean gradient across the prosthetic valve, aortic valve area, indexed effective orifice area, and incidence of moderate and severe patient–prosthesis mismatch.^5^ For the first time, we have found the hemodynamics and left ventricular mass index regression in patients with severe native aortic valve stenosis treated with AVR + Y-AAE are better than those treated transcatheter AVR.

**Figure 1:**
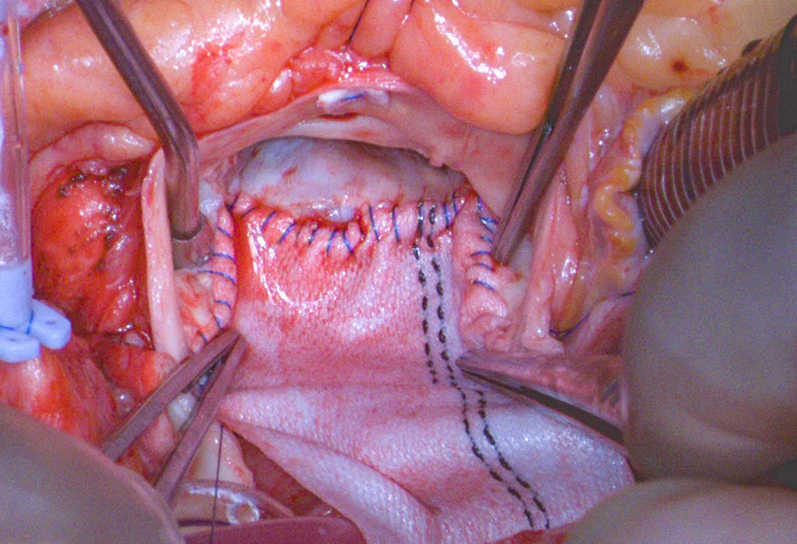
The Y-incision aortic annular enlargement enlarges the surgical aortic annulus/root by enlarging the aortomitral curtain.

The technical key points of the Y-AAE are as follows: (1) make the Y-incision far enough into the left and right fibrous trigones without completely dividing the trigones; (2) use a large patch (3.5 to 4-cm wide) and sew it to the aortomitral curtain but not mitral annulus to prevent a bar underneath the prosthesis (Figure 2, *A*), which could happen when the sewing is flat, such as mechanical prosthesis but hemodynamically insignificant; (3) place the valve sutures on the patch in a dome shape with the highest suture at the same level as the valve sutures at the left-right and right-noncommissures (most times it is about 2 cm away from the bottom suture line of the patch to the aortomitral curtain). The prosthesis is not tilted and does not pinch the right coronary arteries (Figure 2, *B-G*).

**Figure 2:**
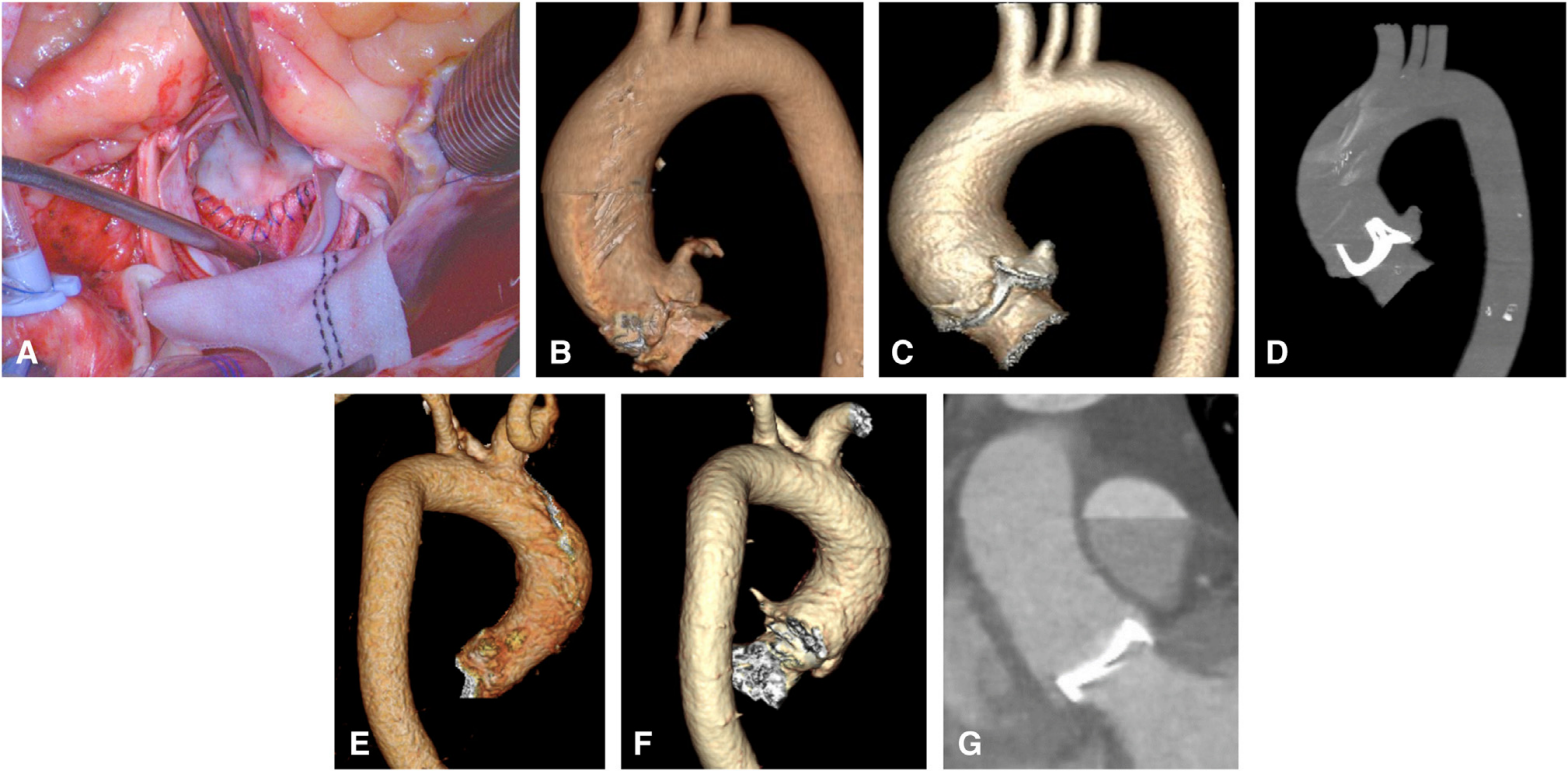
A, A large patch preserved the normal-sized basal ring and allowed a large prosthesis to be implanted, upsizing from a size 23 to size 29. B through D, The surgical aortic annulus was enlarged from 23 mm to a size 29 Magna Ease valve (Edwards Lifesciences). B, Preoperative computed tomography (*CT*) aortogram with 3-dimensional (*3D*) reconstruction. C, Postoperative CT aortogram with 3D reconstruction. D, Postoperative CT aortogram showed the bioprosthetic valve seated well without tilting, and the root and sinotubular junction were enlarged adequately for easy access for coronary artery catheterization and future valve-in-valve transcatheter aortic valve replacement. E through G, The surgical aortic annulus was upsized from 19 mm to size 27 Top Hat (Carbomedics) mechanical valve in a female patient with a body mass index of 53. E, Preoperative CT aortogram with 3D reconstruction, posterior view. F, Postoperative CT aortogram with 3D reconstruction, posterior view showing the Hemashield patch (Getinge). G, Postoperative CT aortogram showed the mechanical valve seated well without tilting.

## Conflict of Interest Statement

Dr Yang is supported by 10.13039/100000002National Institutes of Health grant Nos. R01HL141891 and R01HL151776.

The *Journal* policy requires editors and reviewers to disclose conflicts of interest and to decline handling manuscripts for which they may have a conflict of interest. The editors and reviewers of this article have no conflicts of interest.
